# Discovery of SARS-CoV-2 papain-like protease (PL^pro^) inhibitors with efficacy in a murine infection model

**DOI:** 10.1126/sciadv.ado4288

**Published:** 2024-08-30

**Authors:** Michelle R. Garnsey, Matthew C. Robinson, Luong T. Nguyen, Rhonda Cardin, Joseph Tillotson, Ellene Mashalidis, Aijia Yu, Lisa Aschenbrenner, Amanda Balesano, Amin Behzadi, Britton Boras, Jeanne S. Chang, Heather Eng, Andrew Ephron, Tim Foley, Kristen K. Ford, James M. Frick, Scott Gibson, Li Hao, Brett Hurst, Amit S. Kalgutkar, Magdalena Korczynska, Zsofia Lengyel-Zhand, Liping Gao, Hannah R. Meredith, Nandini C. Patel, Jana Polivkova, Devendra Rai, Colin R. Rose, Hussin Rothan, Sylvie K. Sakata, Thomas R. Vargo, Wenying Qi, Huixian Wu, Yiping Liu, Irina Yurgelonis, Jinzhi Zhang, Yuao Zhu, Lei Zhang, Alpha A. Lee

**Affiliations:** ^1^ Pfizer Global Research and Development, Cambridge, MA 02139, USA.; ^2^ PostEra, 1 Broadway, 14th floor, Cambridge, MA 02142, USA.; ^3^ Pfizer Global Research and Development, Pearl River, NY 10965, USA.; ^4^ Pfizer Global Research and Development, Groton, CT 06340, USA.; ^5^ WuXi, WuXi AppTec (Shanghai) Co. Ltd. Shanghai 200131, China.; ^6^ Pfizer Global Research and Development, La Jolla, CA 92121, USA.; ^7^ Institute for Antiviral Research, Department of Animal, Dairy, and Veterinary Sciences,Utah State University, Logan, UT 84322, USA.; ^8^ Pfizer Global Research and Development, Shanghai 201210, China.

## Abstract

Vaccines and first-generation antiviral therapeutics have provided important protection against COVID-19 caused by severe acute respiratory syndrome coronavirus 2 (SARS-CoV-2). However, there remains a need for additional therapeutic options that provide enhanced efficacy and protection against potential viral resistance. The SARS-CoV-2 papain-like protease (PL^pro^) is one of the two essential cysteine proteases involved in viral replication. While inhibitors of the SARS-CoV-2 main protease have demonstrated clinical efficacy, known PL^pro^ inhibitors have, to date, lacked the inhibitory potency and requisite pharmacokinetics to demonstrate that targeting PL^pro^ translates to in vivo efficacy in a preclinical setting. Here, we report the machine learning–driven discovery of potent, selective, and orally available SARS-CoV-2 PL^pro^ inhibitors, with lead compound PF-07957472 (**4**) providing robust efficacy in a mouse-adapted model of COVID-19 infection.

## Teaser

Orally active SARS-CoV-2 papain-like protease inhibitors demonstrate in vivo efficacy in a murine infection model.

## INTRODUCTION

Coronaviruses are a group of positive-sense single-stranded RNA viruses known to cause human pathologies, ranging from mild to moderate upper respiratory tract illnesses (229E, NL63, OC43, and HKU1) to global outbreaks [severe acute respiratory syndrome coronavirus (SARS-CoV), Middle East respiratory syndrome-CoV, and SARS-CoV-2] (*1*, *2*). In particular, the COVID-19 pandemic caused by SARS-CoV-2 has led to more than 14 million estimated excess deaths worldwide as of late 2023. Highly effective vaccines have provided community-wide protection, while oral antivirals are now approved for adults with mild-to-moderate COVID-19 who are at high risk for progression to severe disease.

Currently, SARS-CoV-2 antivirals, which have been clinically approved or are in late-stage development, are directed against either the RNA-dependent polymerase (RdRp) or the main protease (M^pro^) (*3*). With the possibility of resistance developing over time (*4*–*6*), there is a need for additional oral antivirals directed against currently undrugged viral targets.

The SARS-CoV-2 papain-like protease (PL^pro^) is a cysteine protease and a domain in the coronavirus nonstructural protein 3 (Nsp3). Conserved across coronaviruses, PL^pro^, along with M^pro^, is responsible for polyprotein processing, which is required for the generation of a functional viral replicase (*7*). In addition, PL^pro^ functions as a deubiquitinase/deISGylase and is thought to modulate host innate immune pathways via cleavage of the posttranslational modifications ubiquitin and interferon-stimulated gene 15 (ISG15) from host proteins as an evasion mechanism (*8*). As such, PL^pro^ is hypothesized to be an essential viral enzyme (*9*), and PL^pro^-targeting antivirals may offer a differentiated mechanism of action compared to antivirals in the current therapeutic landscape.

While PL^pro^ inhibitors have been of interest, development has lagged behind the discovery of advanced M^pro^ inhibitors. SARS-CoV-2 M^pro^, also known as the 3C-like protease (3CL^pro^), is also a viral cysteine protease that cleaves the viral polyprotein during replication, acting at 11 cleavage sites as compared to the 3 cleavage sites of PL^pro^. Similarities of the protease-substrate interaction and catalytic cysteine of M^pro^ to that of human rhinovirus 3C protease (3C^pro^) and transmissible gastroenteritis virus 3CL^pro^ aided early discovery of SARS-CoV M^pro^ inhibitors (*10*). These efforts eventually resulted in a development candidate for SARS-CoV, which did not progress through clinical trials due to containment of the prior pandemic (*11*). However, these inhibitors demonstrated the promising antiviral potential of M^pro^ inhibitors and provided key starting points for SARS-CoV-2 antiviral development (*12*).

Unlike M^pro^, there are two key missing elements in understanding the therapeutic potential of targeting PL^pro^: (i) whether inhibition of PL^pro^ can result in cellular antiviral activity at therapeutically meaningful potency and (ii) whether potent PL^pro^ inhibition can translate to in vivo efficacy with compounds that have a well-characterized selectivity profile and favourable pharmacokinetic attributes in preclinical species. In particular, animal disease models of COVID-19 can help establish the translation between in vitro PL^pro^ inhibition and in vivo viral suppression, as well as potentially shed light on the interplay between infection and immune response (*13*). However, identification of PL^pro^ inhibitors suitable for in vivo validation is challenging with respect to achieving the desired combination of the requisite PL^pro^ potency, cellular antiviral activity, and appropriate preclinical pharmacokinetics as maintaining trough plasma concentrations in vivo at multiples of the cellular antiviral potency is necessary (*12*, *14*). Thus far, compounds in the literature include compounds developed for SARS-CoV PL^pro^ (*9*, *15*–*18*), which also show activity against SARS-CoV-2 PL^pro^ (most notably, GRL0617, compound **1**), as well as compounds identified more recently to combat the SARS-CoV-2 pandemic. Although valuable starting points, these molecules have shown only weak cellular antiviral potencies in the low-to-high micromolar range with minimal information on in vitro (or in vivo) disposition characteristics in animals and/or human reagents. Here, we report the machine learning–driven discovery of potent, selective, and orally bioavailable SARS-CoV-2 PL^pro^ inhibitors with robust efficacy in a mouse-adapted model of COVID-19 infection.

## RESULTS

### Discovery of potent PL^pro^ inhibitors

Given the expected need for rapid improvements in relevant physicochemical properties during the rapidly evolving pandemic, a key approach of our medicinal chemistry strategy was data-driven and algorithmic. Artificial intelligence and machine learning have seen increased use in small-molecule drug discovery in recent years (*19*, *20*). As part of our machine learning–augmented approach, we used parallel high-throughput chemistry to rapidly scan large regions of chemical space, probe multiple synthetically accessible vectors, and diversely select compounds for each library based on bioactivity predictions (*21*, *22*). Critical to the success of the collaboration between Pfizer and PostEra to ensure efficiency was to leverage the integration of Contract Research Organization networks as described in fig. S1. The workflow setup allowed for speed in execution and delivery of compounds and data to drive structure-activity relationships.

**Fig. 1.**
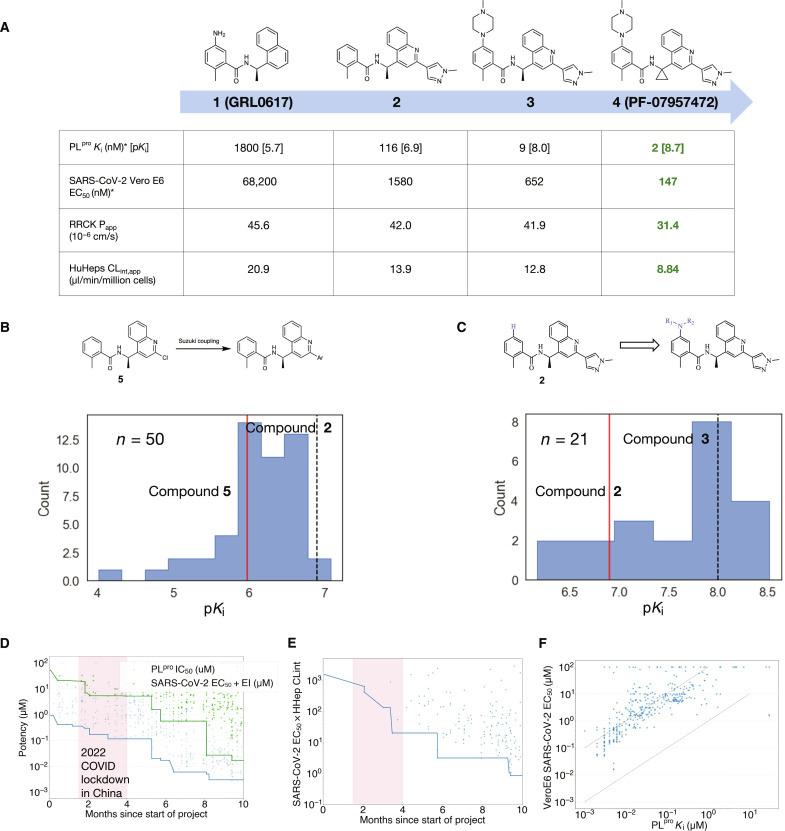
Discovery of PL^pro^ inhibitors guided by machine learning. (**A**) Summary of key compounds in our discovery campaign with their biochemical potency, cell antiviral potency in Vero E6 cells containing the P-glycoprotein (Pgp) inhibitor CP-100356 (2 μM), passive permeability determined by Ralph Russ canine kidney (RRCK) cells (*23*) and apparent intrinsic clearance (CL_int,app_) obtained from metabolic stability studies in cryopreserved human hepatocytes (*24*). *All values provided are the geometric mean of at least three replicates. (**B** and **C**) The potency of compounds made in the Suzuki and C-N coupling libraries; *n* denotes the number of compounds made in each library. The red line denotes the potency of the compound that inspired the library. (**D**) Biochemical (blue) and cell (green) potency over time, and (**E**) product between potency and CL_int,app_ in human hepatocytes (*24*) of synthesized compounds plotted over time. In (D) and (F), each data point is a compound where we track the date of synthesis completion, and the solid lines show the running minimum. As our synthetic chemistry efforts were based in China, COVID lockdown there precipitated a marked disruption to campaign progress. (**F**) PL^pro^ inhibition translates to antiviral activity. *K*_i_ and inhibition are correlated on a cellular level [in the presence of 2 μM of Pgp inhibitor CP-100356 to counteract the high levels of Pgp efflux in Vero E6 cells (*12*)]. The dashed lines show EC_50_ = *K*_i_ and 100 × *K*_i_, respectively.

We started from known compounds such as the widely studied GRL0617 (**1**), a non-covalent inhibitor active against SARS-CoV, subsequently found to be active against SARS-CoV-2 PL^pro^ (Fig. 1A) (*15*, *16*). Compound **1** inhibits recombinant SARS-CoV-2 PL^pro^ with a 1.8 μM inhibition constant (*K*_i_) in our fluorescence resonance energy transfer–based substrate cleavage assay. In a cellular context, **1** showed weak antiviral effects [half-maximal effective concentration (EC_50_) of 68.2 μM] measured by monitoring the cytopathic effect (CPE) in Vero E6 cells in the presence of the P-glycoprotein (Pgp) efflux inhibitor CP-100356 (*12*). To improve upon the existing chemical matter, we identified two regions for optimization: the naphthalene ring system and the aniline substituent. Our first round of medicinal chemistry efforts focused on elaboration of the naphthalene ring. Scanning available building blocks led to the discovery of a quinoline substituent as a suitable replacement. A large Suzuki coupling library (50 compounds) was designed using machine learning to explore the 2-position (Fig. 1B) while balancing predicted bioactivity, molecular diversity, and the synthetic requirements of building blocks for a successful execution of the parallel library chemistry approach. Our library led to the identification of the *N*-methyl pyrazole (**2**) derivative with an order-of-magnitude improvement in PL^pro^ *K*_i_, even in the absence of the aniline functional group.

Our second round of optimization focused on modifying the aniline motif. To this end, we constructed libraries (21 compounds) based on C-N coupling (Fig. 1C), which led to the identification of methyl piperazine substituent (**3**) with another order-of-magnitude improvement in *K*_i_ while also providing a twofold improvement in viral CPE potency in the Vero E6 assay. Last, rigidification of the scaffold via introduction of a geminal-cyclopropyl group in lieu of the pendant methyl substituent resulted in the lead compound (PF-07957472, **4**) with an additional threefold improvement in biochemical and cellular antiviral potencies and also a reduction in the apparent intrinsic clearance (CL_int,app_) in a metabolic stability assay using human hepatocytes (*23*).

This strategy led to a compressed timeline (Fig. 1D) wherein month-on-month improvements in biochemical potency against PL^pro^ and cellular antiviral activity were achieved. With a desire to establish proof of mechanism in an animal model of COVID-19 and potentially identify chemical lead matter with clinical candidate-like qualities, a crucial indicator of campaign progress was the concomitant optimization of potency and metabolic stability of new lead compounds. This balance could be captured by evaluating the product between cellular antiviral activity (EC_50_) and metabolic CL_int,app_ estimated in human hepatocyte incubations, a quantitative score inspired by a fit-for-purpose human dose prediction (see the “Methodology for multi-parameter optimization scoring” section in the Supplementary Materials) (*24*). The quantitative score revealed a steady reduction over time (Fig. 1E), showing that desired PL^pro^ inhibitory potency and cellular antiviral activity could be achieved for compounds with reasonably low CL_int,app_ values in human hepatocytes. Additional confidence in PL^pro^ as an antiviral target was evident from the strong correlation between enzymatic inhibition of PL^pro^ and cellular antiviral activity (Fig. 1F). In general, the cellular antiviral EC_50_ was observed to be approximately two orders of magnitude weaker than the corresponding biochemical *K*_i_ against recombinant PL^pro^. Although the precise reasons for this drop-off remain elusive, the direct correlation between biochemical and cell potency and the attainment of low nanomolar cell potency provide a path to achieving potent antiviral compounds.

### Characterization of PF-07957472 as an in vivo tool compound

**Fig. 2.**
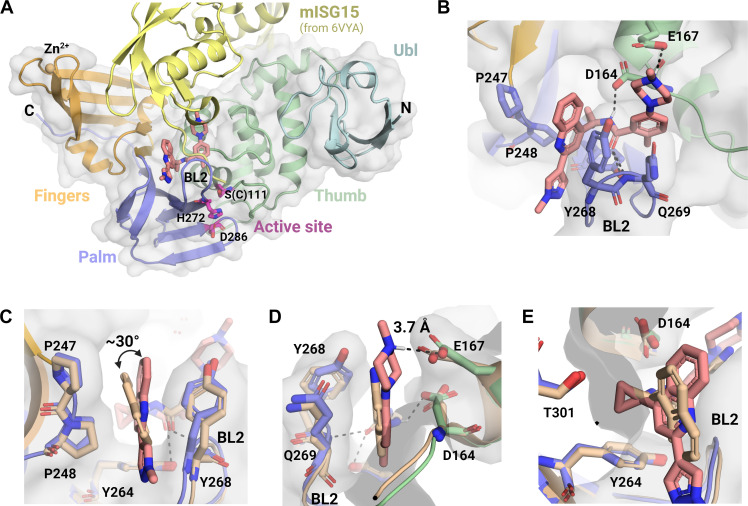
X-ray crystal structure of compound 4 bound to SARS-CoV-2 PL^pro^ and structural comparison with GRL0617 binding. In each panel, SARS-CoV-2 PL^pro^ bound to **4** (sticks, salmon) is shown in cartoon representation, color-coded by subdomain, and with light gray surface representation. (**A**) Structural superimposition of SARS-CoV-2 PL^pro^ bound to **4** and SARS-CoV-2 PL^pro^ bound to mouse ISG15 [yellow; Protein Data Bank (PDB) ID 6YVA]. The PL^pro^ protein structure in 6YVA is not shown for clarity. The C terminus of ISG15 (His^149^-Gly^155^) overlaps with the binding site of **4**. The catalytic triad is shown in magenta; a catalytically dead construct of PL^pro^ (C111S) was used to generate the crystal structure. (**B**) Detailed view of **4** binding in the BL2 loop of SARS-CoV-2 PL^pro^, highlighting critical protein-ligand interactions. Hydrogen bonds are represented as black dashed lines. Tyr^264^ is not labeled but is in the background, forming a hydrogen bond with the amide of **4** (**C** to **E**) Structural overlays of SARS-CoV-2 PL^pro^ bound **4** and SARS-CoV-2 bound to GRL0617 (wheat; PDB ID 7CMD). The orientation of the binding pocket varies in each panel, with the BL2 loop labeled throughout. Surface representation is shown for the structure of SARS-CoV-2 PL^pro^ bound to **4** only.

A crystal structure of SARS-CoV-2 PL^pro^ in complex with **4** was determined to 2.59 Å (table S2). This structure reveals that **4** engages a region on PL^pro^ that overlaps with the substrate binding site but does not extend to the catalytic triad active site (Fig. 2A). Compound **4**, like GRL0617, binds in the pocket formed by the flexible BL2 loop and forms critical hydrogen bonds with Asp^164^, Glu^167^, Tyr^264^, and the backbone nitrogen of Gln^269^, as well as π stacking interactions with Pro^247^, Pro^248^, and Tyr^268^ (Fig. 2B) (*16*, *25*). The binding pose assumed by **4** differs from that of GRL0617 in the following ways: (i) the *N*-methyl pyrazole-quinoline aromatic system in **4**, in place of the naphthalene moiety, gains greater surface area coverage of the hydrophobic “shelf,” formed by Pro^247^ and Pro^248^, and more efficient T-shaped π-stacking interactions with Tyr^268^ from a ~30° tilt at the quinoline core toward this residue (Fig. 2C); (ii) the protonated piperazinyl *N*-methyl amine engages Glu^167^ in a salt bridge (3.7 Å), while the analogous primary amine of the aniline of GRL0617 is 6 Å away from Glu^167^ (Fig. 2D); and (iii) the cyclopropyl is a larger hydrophobic substitution that better engages Tyr^264^ via CH-π interactions, while its hydrogen atoms, polarized due to conformational strain, can also interact with the polar residues in the pocket such as Thr^301^ and Asp^164^, a feature that is missing in GRL0617, which has a smaller methyl group at this position (Fig. 2E). These optimized protein-ligand interactions rationalize the greatly improved potency observed for **4**, compared to GRL0617 (Fig. 2, C to E).

**Fig. 3.**
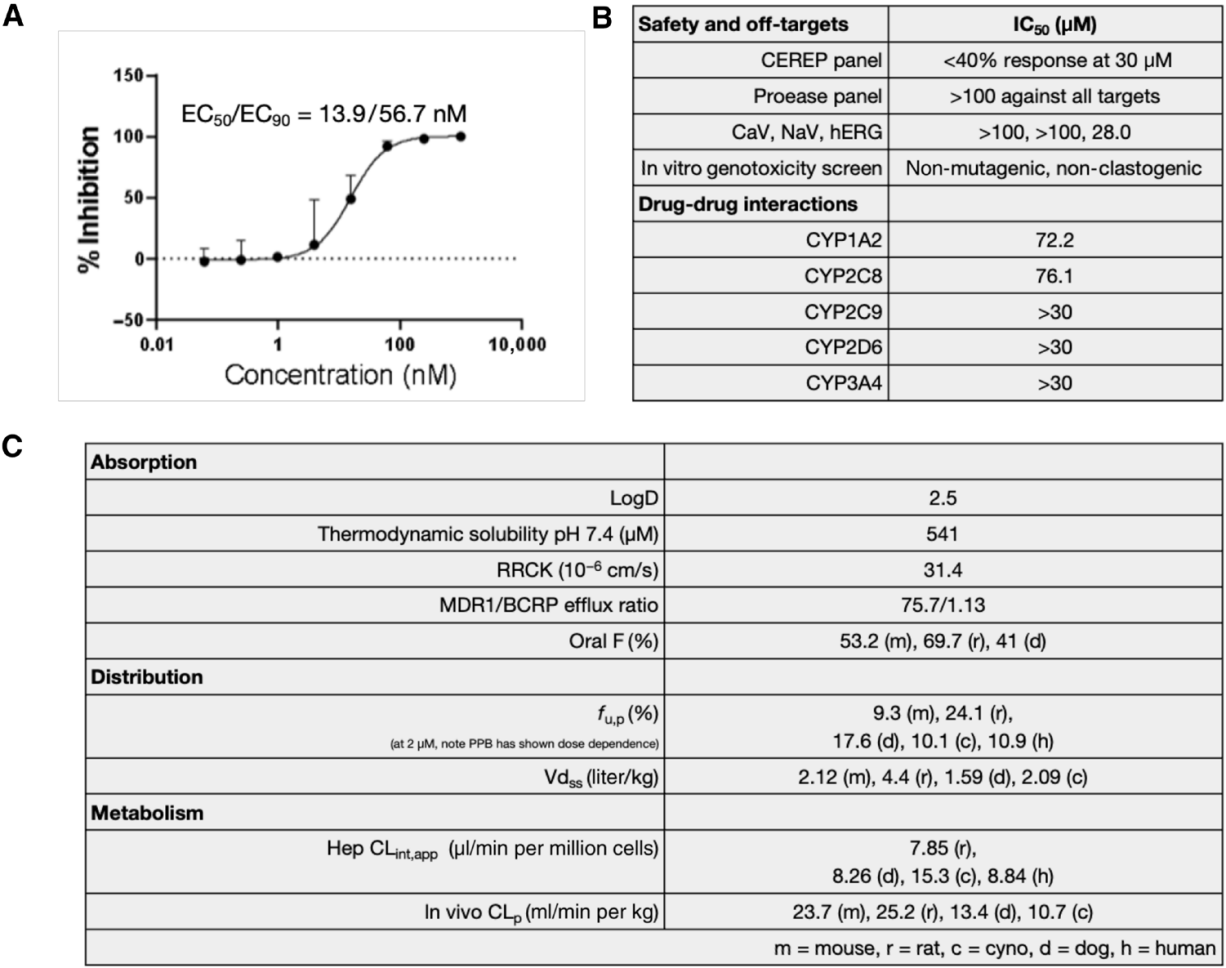
Compound 4 is an antiviral lead and a suitable in vivo tool compound. (**A**) Dose-response curve of **4** assayed in a human airway epithelial cell model of SARS-CoV-2 infection (*12*). The fraction unbound of **4** in the dNHBE medium was measured to be 0.916, assumed to be 1 for modeling purposes. (**B**) Profiling **4** through safety panels and CYP panels reveal no clear off-target liabilities. See table S1 for full Cerep profile. (**C**) In vitro and in vivo absorption, distribution, and metabolism properties across multiple species. MDR, multi-drug resistance protein 1 (P-glycoprotein) efflux ratio; BCRP, murine Breast Cancer Resistance Protein BA/AB ratio; PPB, Plasma Protein Binding.

We profiled **4** in a primary human airway epithelial [differentiated normal human bronchial epithelial (dNHBE)] cell infection model, a physiologically relevant system that had shown better in vitro–in vivo correlation than Vero E6 cells to the observed efficacy with published M^pro^ inhibitors (*26*). Compound **4** demonstrated potent viral CPE in dNHBE cells (EC_50_ = 13.9 nM) (Fig. 3A and fig. S3). To identify potential off-target activity, we profiled **4** through standard safety pharmacology panels as well as comprehensive protease panels (further details in tables S1 and S3). We detected no major off-target activity, with only moderate human ether-a-go-go (hERG) potassium ion channel (product of the hERG gene; Kv11.1) activity [median inhibitory concentration (IC_50_) = 28.0 μM], which was considered marginal, given the potent antiviral activity in cells. Compound **4** was devoid of potent reversible inhibition of major cytochrome P450 (CYP) isoforms, including the major human constitutive CYP enzyme CYP3A4 (Fig. 3B). The observation that selective PL^pro^ active site inhibition leads to low nM antiviral activity in a primary cellular system provides additional evidence for the essentiality of PL^pro^ in viral replication.

Compound **4** was shown to have favorable in vitro ADME properties including high thermodynamic solubility and passive permeability in the Ralph Russ canine kidney (RRCK) assay and a low metabolic CL_int,app_ in human hepatocytes (Fig. 3C). Following intravenous administration to preclinical species (mice, rats, dog, and monkeys), **4** demonstrated moderate plasma clearances (CL_p_) across species and high steady-state distribution volumes (Vd_ss_). Despite being a Pgp substrate (*27*), **4** demonstrated moderate oral bioavailability (*F*) and a high fraction of the oral dose absorbed across the preclinical species studied, in a relatively straightforward formulation comprising of 0.5% methylcellulose in water containing 2% Tween 80. The oral pharmacokinetics of **4** were encouraging, particularly against the backdrop of established pharmacokinetic-pharmacodynamic relationships for SARS-CoV-2 protease inhibitors [and protease inhibitors for HIV and hepatitis C virus as well; (*14*)], which require trough or minimum plasma concentrations (*C*_min_) to be maintained above cellular EC_90_ during the entire treatment duration (*3*).

### PL^pro^ inhibitor PF-07957472 in a murine SARS-CoV-2 infection model

Given its promising antiviral activity and acceptable rodent oral F, a multidose mouse pharmacokinetic study was conducted with orally administered **4** (30, 150, and 500 mg/kg), which demonstrated that unbound systemic exposures were considerably higher than the dNHBE EC_90_ of 56.7 nM. Moreover, **4** was tolerated at the highest dose studied.

On the basis of the findings from the preliminary dose-range finding studies, antiviral activity of **4** was examined in a mouse-adapted SARS-CoV-2 (SARS-CoV-2 MA10) model (*28*), following twice daily (BID) oral administration at 20, 50, and 150 mg/kg, corresponding to intended unbound *C*_min_ margins of ^1^/_3_×, 1×, and 3× EC_90_. Nirmatrelvir alone was included as a positive control and dosed orally at 1000 mg/kg (unbound *C*_min_ ~ 4× EC_90_, BID), which had proven to be efficacious in this mouse model (*12*).

**Fig. 4.**
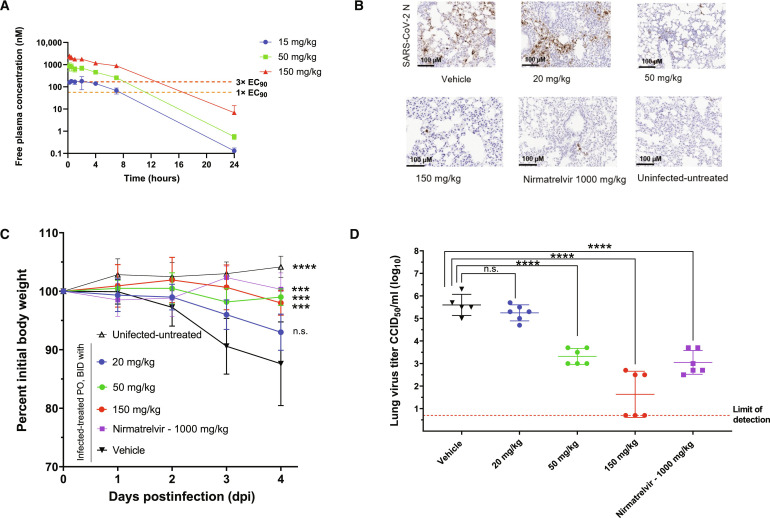
PL^pro^ inhibitor 4 is efficacious in a murine SARS-CoV-2 infection model. (**A**) Dose-range finding studies showed that the selected doses cover a range of trough concentrations from subtherapeutic to therapeutic levels. (**B**) Representative immunohistochemistry images from lung histopathology of mouse-adapted SARS-CoV-2 infection 4 days after dose of **4** at 20, 50, and 150 mg/kg; Nirmatrelvir at 1000 mg/kg; vehicle; or uninfected, untreated animals. (**C**) Compound **4** protected mice from weight loss, with roughly 10% weight loss seen in the vehicle arm consistent with data in (*12*). PO, by mouth/oral administration; ***P < 0.001; ****P < 0.0001; n.s., not significant. (**D**) Compound **4** led to a statistically significant and dose-dependent reduction in day 4 lung viral titers. Titers were plotted as mean log_10_ 50% cell culture infectious dose (CCID_50_)/ml ± SEM, with data analysis and significance levels matching those in (*12*).

In the efficacy study, mice were infected 4 hours before the first dose, dosed BID for 4 days, and then viral lung titers were measured. Compound **4** caused statistically significant reduction in lung viral titers at 4 days postinfection (dpi) for both dose groups at 50 and 150 mg/kg at unbound systemic exposures (*C*_min_) that maintained or exceeded the dNHBE EC_90_ through the dosing period (Fig. 4, A, B, and D). The observed efficacy is comparable to that observed with the SARS-CoV-2 M^pro^ inhibitor Nirmatrelvir (Fig. 4, B and D). While infected and untreated mice had robust infection at day 4, half of the mice in the dose at 150 mg/kg had viral levels reduced to the limit of viral detection. In addition, BID treatment with **4** protected mice from weight loss compared with the infected mice in the vehicle group, which showed ~10% body weight loss following infection as expected (Fig. 4C). Overall, these findings confirmed that PL^pro^ inhibition is effective at reducing SARS-CoV-2 viral replication in mouse lungs and that the PL^pro^ inhibitor **4** is an effective in vitro and in vivo tool compound for further studies of this mechanism of antiviral effect.

### Analysis of naturally occurring PL^pro^ mutants and PL^pro^ homologs with PF-07957472

**Fig. 5.**
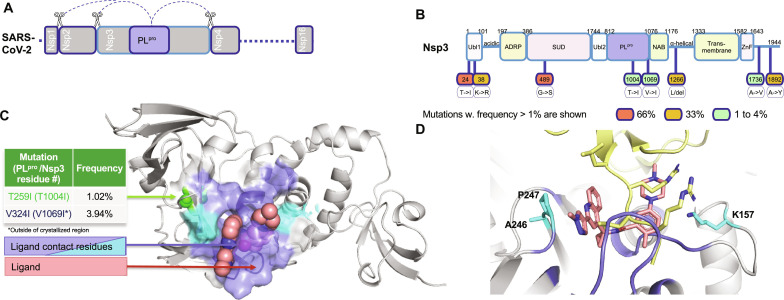
High-frequency mutations in Omicron variants (6,891,950 reads) on Nsp3 and resistance selection mutants of PL^pro^. (**A**) The location of the cysteine protease, PL^pro^ in SARS-CoV-2 genome, and the cleavage sites that it processes to generate functional viral proteins. (**B**) The Nsp3 protein (residues 1 to 1944) showing all domains identifying clinically observed Omicron mutations with frequency > 1%. (**C**) PL^pro^ domain (residues using PL^pro^ numbering 3 to 315) with location of mutation with frequency > 0.5% in green, which are distal from residues within 5.5 Å for **4** (pink spheres). Binding surface is shown in as blue and cyan, where cyan indicates the three highest frequency mutations (A246, P347, and K157) within the ligand binding site (table S8). (**D**) Structural superposition of ISG15 binding site and **4** showing interaction with similar residues.

Concomitant to our discovery efforts, we used publicly available sequencing information from the Global Initiative on Sharing All Influenza Data to understand the mutational profile of the PL^pro^ domain to assess its viability for drug discovery. PL^pro^ is one of the eight structured domains on Nsp3 and is not cleaved as an independent protein (Fig. 5, A and B). For this reason, we examined the mutational frequency of all residues on Nsp3, encompassing 1944 residues, with a focus on those with mutation frequency ≥1%. This work identified eight mutations in Nsp3 (Fig. 5B), and the two most frequent mutations were found in the ubiquitin-like domain 1 (Ubl1) and SARS-unique domain (SUD) domains (~66% mutation rate for both T24I and G489S). Taking a closer look at the PL^pro^ domain-specific mutations, only three naturally occurring mutations were identified with a frequency > 0.5%: P985S (0.52%), T1004I (1.02%), and V1069I (3.94%) (Fig. 5C), all of which are outside the inhibitor binding pocket (frequencies in table S8). This provides good confidence that this is a stable binding pocket and that **4** would be a stable and effective molecule against SARS-CoV-2 infections. This finding is expected as native substrates of PL^pro^ such as ISG15 overlap with the inhibitor binding pocket (Fig. 5D).

Activities of **4** were profiled against several PL^pro^ proteins from other viruses. Compound **4** was equally potent on SARS-CoV but nearly inactive on other viruses like MERS, 229E, and OC43 (table S3), suggesting that differences in the binding pocket and especially residues in the BL2 loop (fig. S2) that make key interactions with the inhibitor could prevent ligand binding and cause loss of inhibitor fidelity.

## DISCUSSION

We disclose the discovery of potent, selective, and orally available PL^pro^ inhibitors that show potent cellular antiviral activity and in vivo efficacy. Our results demonstrate that PL^pro^ is an essential and druggable antiviral target. Further, the pharmacokinetics and in vitro safety profile of our lead compound PF-07957472 (**4**) show that it is a valuable in vitro and in vivo tool for further study of PL^pro^-targeted therapeutics.

Given that PL^pro^ inhibitors offer a differentiated mechanism from existing SARS-CoV-2 antivirals, an extension of this work would be the study of these compounds in combination with SARS-CoV-2 M^pro^ or RdRp inhibitors. Despite the robust single-agent efficacy observed in this study, a combination approach may potentially offer additional benefit in a clinical setting. As PL^pro^ is also thought to modulate host innate immune pathways, an open question is the degree of benefit that this immunomodulatory effect confers in an in vivo setting. Further, characterization of our inhibitors in passaging studies is needed to understand the barrier to resistance associated with PL^pro^ inhibition.

More broadly, our work highlights the utility of machine learning–enabled medicinal chemistry. Through judicious use of parallel medicinal chemistry libraries, we were able to progress the campaign steadily, leading to the discovery of a compound with robust in vivo efficacy in less than 8 months of medicinal chemistry efforts.

## MATERIALS AND METHODS

### In vitro pharmacology

#### PL^pro^ enzymatic assay

The compounds were tested in a PL^pro^ biochemical assay to determine their ability to inhibit the enzyme’s activity. Biochemical potencies are reported as the concentration of the compound required to achieve 50% inhibition of the enzyme activity in the assay (IC_50_). *K*_i_ values were fit to the tight binding Morrison equation with fixed parameters for enzyme concentration, substrate concentration and the Michaelis constant (*K*_m_) parameter.

The potency of compounds against the SARS-CoV-2 PL^pro^ was measured using a synthetic profluorogenic substrate, Z-RLRGG-AMC (GenScript). Compounds were serially diluted more than 11 points using 100% dimethyl sulfoxide (DMSO) as a diluent, from either a top concentration of 3 mM with a half-log dilution factor or a top dose of 0.1 mM with a twofold dilution factor. The final top dose of compounds in the assay was either 30 or 1 μM (1% DMSO). The assay buffer contained 50 mM Hepes (pH 7.5), 1 mM tris(2-carboxyethyl)phosphine (TCEP), and 0.1% bovine serum albumin. Final assay conditions included 6.25 nM PL^Pro^ enzyme and 25 μM peptide substrate in 1% DMSO, with an enzyme reaction time of 60 min.

Briefly, 250 nl of serially diluted compound was spotted into a white 384-well plate, followed by addition of SARS-CoV-2 PL^pro^ enzyme (7.8 nM, 20 μl) in assay buffer. The compounds and PL^pro^ enzyme were preincubated for 30 min at room temperature. The reaction was then initiated by the addition of profluorogenic peptide substrate in assay buffer (5 μl, 125 μM Z-RLRGG-AMC). The reaction was allowed to progress for 60 min at 25°C after which the plate was read on a Molecular Devices Spectramax M2e reader at an excitation/emission of 360/460 nm. The no-compound, zero percent inhibition (ZPE) control wells contained 1% DMSO with substrate and PL^pro^ enzyme. The hundred percent effect (HPE) wells contained an internal Pfizer control compound at a dose sufficient to accomplish complete inhibition (1% DMSO), substrate, and PL^pro^ enzyme. Data were analyzed with ActivityBase software (IDBS Ltd.). The raw data were transformed to percent activity values using the average from the ZPE and HPE control wells. The resulting data were fit with the four-parameter logistic fit model to determine the IC_50_ value. For compounds eliciting high potencies, the percent activity values can be fit to the Morrison equation to derive *K*_i_ values with the following fixed parameters: enzyme concentration, 6.25 nM; substrate *K*_m_, 962 μM; and substrate concentration, 25 μM. To qualify inter-experimental performance, the internal control (R)-5-(aminomethyl)-2-methyl-*N*-(1-(naphthalen-1-yl)ethyl)benzamide [compound 2, c.f. (*29*)] was tested in each run.

#### Cellular antiviral activity

The ability of compounds to inhibit viral induced CPE against human coronaviruses was measured using the VeroE6 assay with CPE and cytotoxicity endpoints using the procedure described in (*12*).

Select compounds were also evaluated using SARS-CoV-2 strain USA-WA1/2020 (BEI Resources, catalog no. NR-52281) in dNHBE cells (MatTek Corporation, Ashland, MA). The cells were grown in MatTek’s proprietary culture medium (AIR-100-MM) according to manufacturer’s protocol and infected in a biosafety level 3 facility as described by Owen *et al.* (*12*). Briefly, both virus and compound were diluted in AIR-100-MM medium. Cells were infected at an approximate multiplicity of infection of 0.001 for 2 hours. Inhibitor was serially diluted in medium from 1 to 0.06 μM. Compound and virus in medium were applied to the apical side, whereas compound and medium only were added to the basal side. Following 2 hours of infection, the apical medium was removed and washed twice with phosphate-buffered saline (PBS), and fresh compound in medium was added to the basal side. At 3 dpi, mucus was collected from the apical side, and virus was quantified by plaque assay adapted from Natekar *et al.* (*30*). Briefly, infected Vero E6 cells (American Type Culture Collection, catalog no. CRL-1586) were overlayed with semisolid overlay medium [0.6% Tragacanth-Gum (Sigma-Aldrich, catalog no. G1128-100G) and 2× Dulbecco’s modified Eagle’s medium (Gibco, catalog no. 11935046)] and detected using crystal violet (1% w/v) in 20% ethanol after 72 hours of incubation. To determine the EC_50_ and EC_90_, the values of plaque-forming units per milliliter were normalized to that of no-drug control as a percentage of inhibition and plotted against compound concentration in GraphPad Prism software by using four-parameter logistic regression.

#### Bacterial reverse mutation assay

The mutagenic potential of PF-07957472 was evaluated using the protocol described in (*12*). PF-07957472 was not mutagenic or clastogenic in all tested in vitro genetic toxicity studies.

### In vivo pharmacology

All procedures performed on animals were in accordance with regulations and established guidelines and were reviewed and approved by an Institutional Animal Care and Use Committee or through an ethical review process (number 11151).

#### Mouse-adapted SARS-CoV-2 infection and treatment studies

The in vivo infection studies were performed in an animal biosafety level 3 facility in the Association for Assessment and Accreditation of Laboratory Animal Care–accredited Laboratory Animal Research Center at Utah State University. Pharmacokinetic studies were performed in an animal biosafety level 2 facility. The study procedures were conducted with approval by the Institutional Animal Care and Use Committee at Utah State University. A total of 48 BALB/c mice (Charles River Laboratories, 8-week-old female) were divided into six groups as follows: untreated, uninfected (mock) (*n* = 6); untreated infected (vehicle) (*n* = 6); PF-07957472 at 20 mg/kg (*n* = 6); PF-07957472 at 50 mg/kg (*n* = 6); PF-07957472 at 150 mg/kg (*n* = 12); and Nirmatrelvir (1000 mg/kg; *n* = 12). Another satellite group of six mice consisting of two mice per dose of PF-07957472 was treated with indicated doses (PF-07957472 at 20, 50, and 150 mg/kg and Nirmatrelvir at 1000 mg/kg), and plasma were collected at 1, 3, 6, and 12 hours after treatment for pharmacokinetic analysis. For infections, mice were anesthetized by intraperitoneal injection of ketamine/xylazine (50/5 mg/kg) and inoculated intranasally with 1 × 10^5^ 50% cell culture infectious dose (CCID_50_) of SARS-CoV-2 MA10 (90 ml per naris). The mouse-adapted MA10 virus was provided by R. Baric (University of North Carolina) (*28*). For oral administration, PF-07957472 was solubilized in 2% (v/v) Tween 80 in 98% (v/v) of 0.5% (w/v) methylcellulose in deionized water by geometric dilution. Mice were dosed BID × 4 days beginning at 4 hours after infection. Mice were weighed daily starting at day 0 until end of study to measure infection-associated weight loss. At 4 dpi, mice were euthanized by isoflurane inhalation. The lungs were collected and placed in 1 ml of PBS and stored at −80°C for evaluation of lung virus titers or collected for histopathology as described below. For virus titer assays, serial log_10_ dilutions of 1.0 ml of lung tissue homogenates were performed in quadruplicate on confluent monolayers of Vero E6 cells seeded in 96-well microplates. The cells were incubated at 37°C and 5% CO_2_ for 6 days and then scored for CPE using a light microscope. Virus lung titer [CCID_50_/ml (log_10_)] was calculated by linear regression using the Reed-Muench method (*31*).

#### Lung immunohistochemistry assessment

For immunohistochemistry staining of SARS-CoV-2 nucleocapsid protein, 4-mm sections were obtained from formalin-fixed paraffin-embedded lung tissue and immunostained using the Leica Biosystems Bond automated stainer (performed at HistoWiz Inc., Brooklyn). Epitope retrieval was performed using citrate-based pH 6 solution for 20 min at 95°C for heat-induced epitope retrieval. The tissue sections were then incubated with background eraser blocking reagent (Leica Biosystems) for 10 min to prevent nonspecific binding. Next, the tissue sections were incubated for 30 min with SARS-CoV-2 (COVID-19) nucleocapsid antibody (HL448; GTX635686, GeneTex) at a 1:10,000 dilution, followed by incubation for 30 min with DAB (3,3’-diaminobenzidine) rabbit secondary reagents (Bond Polymer Refine Detection, DS9800, Leica Biosystems). The slides were visualized using an Aperio AT2 slide scanner (Leica Biosystems).

#### Lung histopathology assessment

To assess virus-induced damage to the lungs of SARS-CoV-2 MA10-infected mice, mice were euthanized at 4 dpi, and lung lobes were collected for virus titer evaluation or left lobes were fixed in 4% paraformaldehyde at 4°C for histopathology (*12*). Fixed lung lobes were shipped to an external histology laboratory (HistoWiz Inc.) for processing and blinded evaluation by an experienced veterinary pathologist and yielded similar results. Group samples (*n* = 6) were processed as one hematoxylin and eosin–stained slide from each lung specimen. Each lung sample was evaluated using a semiquantitative analysis using four parameters: perivascular inflammation, bronchial or bronchiolar epithelial degeneration or necrosis, bronchial or bronchiolar inflammation, and alveolar inflammation. A five-point scoring system for assessment of epithelial degeneration/necrosis and inflammation was utilized (0, with normal limits; 1, mild: scattered cell necrosis/vacuolation and few/scattered inflammatory cells; 2, moderate: multifocal vacuolation or sloughed/necrotic cells and thin layer of inflammatory cells; 3, marked: multifocal/segmental necrosis, epithelial loss/effacement, and thick layer of inflammatory cells; and 4, severe: coalescing areas of necrosis, parenchymal effacement, and confluent areas of inflammation). A total pathology score was calculated for each mouse by adding the individual histopathological scores.

#### Statistics and figures

All graphs were generated using GraphPad Prism. The statistical analysis was performed as follows. For the body weight, the % initial data from days 1 to 4, each dose group was compared to placebo group (PF-09757472, 0 mg/kg) via mixed model analysis. Raw (unadjusted) *P* values are reported. For the lung virus log_10_ titer and histopathology score data, each dose group was compared to that of the group at 0 mg/kg via one-way analysis of variance (ANOVA). Raw (unadjusted) *P* values are reported.

### Synthetic chemistry

See the Supplementary Materials for detailed description of syntheticmethods.

### Machine learning approaches to library design

Our library design strategy uses historic bioactivity data to inform which building blocks are bioisosteric to the starting point. Public historical bioactivity data are processed following (*32*): In summary, congeneric series from the literature can be extracted by mining ChEMBL (*33*), applying the requisite filter to ensure that these series come from the same assay and publication. These congeneric series are then fragmented into the core and R-groups, allowing us to extract a dictionary of R-groups and activities.

Following (*34*), we then use this dataset to train a neural network classification model to predict, given a particular R-group and the core, which other R-groups or cores are likely to also be active within a series. The trained classification model can then be applied to monomers of parallel medicinal chemistry libraries. First, a reaction disconnection is chosen, along with the reactant for which bioisosteres are desired. The monomers are then cut recursively into R-groups of varying sizes using the fragmentation algorithm described in (*32*). Each R-group is then a candidate for replacement by one of the neural network proposed bioisosteres, and the fragmented molecule is joined together into new monomers using the new R-groups.

After construction of the building blocks, they are checked for synthetic feasibility in the given library chemistry disconnection, and any potential selectivity issues in the reaction are flagged. Furthermore, all candidate products are checked for the presence of new medicinal chemistry alerts introduced by the bioisosteric building block, including those enumerated in sets of alerts created for nuisance compound functionality in compound screening sets and those may be risks for bioactivation (*35*). Last, the candidate building blocks are automatically checked for availability against the ordering constraints supplied by the chemist, including lead time, price, and vendor location. This methodology forms the basis of library design in Fig. 1 (B and C). Further discussion of the library design interface is included in the Supplementary Materials.

## Supplementary Materials

### This PDF file includes:

Supplementary Text Figs. S1 to S4 Tables S1 to S9 References
